# Impact of single nucleotide polymorphism of IL-27P28 rs153109 and IFITM3 rs12252 on susceptibility and severity of COVID-19 in Egyptian patients: a case control study

**DOI:** 10.1186/s12985-025-02668-z

**Published:** 2025-03-08

**Authors:** Hanan Hamdy, Reem H. Elhamammy, Manal Abdelmageed, Ahmed Wahid

**Affiliations:** 1 Faculty of Pharmacy, Alexandria University, Alexandria, 21521 Egypt; 2 Department of Biochemistry, Faculty of Pharmacy, Alexandria University, Alexandria, 21521 Egypt; 3 Department of Experimental and Clinical Internal Medicine, Medical Research Institute, Alexandria University, Alexandria, 21561 Egypt

**Keywords:** SARS-CoV-2, Single nucleotide polymorphism, Interleukin-27, IFITM3 protein, Egyptian people

## Abstract

**Background:**

Severe acute respiratory syndrome coronavirus 2 (SARS-CoV-2) causes Coronavirus disease 2019 (COVID-19), which is a huge global health threat. Interleukin27 (IL-27) gene is a cytokine that produces antiviral proteins in an IFN-independent manner and stimulates both pro- and anti-inflammatory responses. Interferon induced transmembrane protein 3 (IFITM3) inhibits SARS-CoV2 infection by blocking SARSCoV-2 spike proteins which facilitate viral entrance and cell-to-cell fusion. The association between genetic variants and COVID-19 in Egyptians is still unclear. Hence, we sought to investigate the impact of the single nucleotide polymorphism of IL-27P28 rs153109 and IFITM3 rs12252 on the susceptibility and severity of SARS-CoV-2 in Egyptian patients.

**Methods:**

Our study included 242 SARS-CoV-2 patients were recruited from Main University Hospital, Alexandria University, Egypt, and 187 healthy controls. We subdivided the patient group into two subgroups: group A comprised mild/moderate cases (N = 42) (17.4%), and group B included severe/critical cases (N = 200) (82.6%). Genomic DNA was extracted from blood samples using the QIAamp DNA Blood Mini kit, then the PCR products of IL27 and IFITM3 were cut by FastDigest XhoI and MScI, respectively, for detection of SNPs of IL-27P28 rs153109 (-964A/G) and IFITM3 rs12252 (T>C).

**Results:**

The present study found a significant association between IL27 rs153109 (-964A/G) and SARS-CoV-2 infection susceptibility after adjusting for the risk factor (advanced age), IL27 rs153109 (-964A/G) AG genotype (OR = 2.791, 95% CI: 1.237–6.295, *P* = 0.013), AA genotype (OR = 2.385, 95% CI: 1.075–5.291, *P* = 0.033), and (AG+AA vs. GG) genotypes (OR = 2.558, 95% CI: 1.186–5.517, *P* = 0.017). On the other hand, the IFITM3 rs12252(T>C) CT genotype (OR = 1.419, 95% CI: 0.843–2.391, *P* = 0.188), CC genotype (OR = 2.132, 95% CI: 0.436–10.415, *P* = 0.350), and (C/T+C/C vs. TT) genotypes (OR = 1.466, 95% CI: 0.884–2.432, *P* = 0.138) did not show a statistically significant association with either susceptibility or the severity of SARS-CoV-2.

**Conclusion:**

IL27P28 rs153109 AG and AA genotypes of IL27 may be associated with the susceptibility of SARS-CoV-2 infection but not the severity. Concerning the IFITM3 rs12252 SNP, we could not confirm its influence on either susceptibility or the severity of SARS-CoV-2 in this Egyptian population.

## Background

Severe acute respiratory syndrome coronavirus 2 (SARS-CoV-2) is the virus that causes Coronavirus Disease 2019 (COVID-19). This beta coronavirus is linked to severe acute respiratory syndrome coronavirus (SARS- CoV) and Middle East respiratory syndrome coronavirus (MERS-CoV), based on genetic similarity [1].Worldwide, the total cumulative number of SARS-CoV-2 cases and deaths reported to world health organization (WHO) until 16 January 2025 was 777,126,421 and 707,992, respectively [2]. In Egypt, 516,023 confirmed cases and 24,830 deaths were reported to WHO until 16 January 2025 [3]. The severity of SARS-CoV-2 varies greatly among affected persons. Many are asymptomatic; some have mild to moderate symptoms, including dry cough, fever, headache, shortness of breath, malaise, muscle and bone aches. Less common symptoms include productive cough, sore throat, diarrhea, nausea, confusion, hemoptysis, and chest pain, with a large percentage of serious cases developing pneumonia and respiratory distress requiring mechanical ventilation, ICU admission, multiorgan failure, and even death [4].It is suspected that the variable clinical outcomes of SARS-CoV-2 are due to many factors, including viral load, patient comorbidity, advanced age, and host genetic factors like polymorphisms in critical genes [5].

Interleukin 27 (IL-27) gene is located on chromosome 16p11. It is a type 1 cytokine that is part of the IL-6/IL-12 family [6]. It is made up of IL27p28 and Epstein–Barr virus-induced 3 (EBI3) subunits [7]. The major source of IL-27 is produced by stimulated antigen presenting cells (APC) such as macrophages and dendritic cells, which are activated by inflammatory mediators or microbial agents [6]. This cytokine produces antiviral proteins in an IFN-independent manner and stimulates both pro- and anti-inflammatory responses [8]. The single nucleotide polymorphism A964G (rs153109) is a functional polymorphism that occurs at 964 bp upstream of the transcription site of the IL-27 gene and consists of the transition of A to G. This transition causes the development of a novel binding site in the IL-27 gene promoter, which alters the IL-27 gene expression pattern [9]. It was found that IL-27p28 (-964 A>G) (rs153109) SNP; the AA genotype or A allele increases the susceptibility to asthma among the Korean population [10]. The study of the effect of IL-27p28 (-964A/G) on allergic rhinitis in a Chinese Han population found that AA genotypes and the A allele significantly increased the risk of allergic rhinitis, but the AG, GG genotypes and the G allele decreased the risk of allergic rhinitis [11]. Huang et al. [12] found that subjects with the IL-27 rs153109 AG genotype had a 2.22-fold decreased risk of chronic obstructive pulmonary disease compared with the control group among the Chinese population.

Interferon-induced transmembrane (IFITM) proteins are encoded by the IFITM gene located on chromosome 11p15.5. These proteins are constitutively expressed in a variety of cell types. They play a key role in adaptive immunity by inhibiting viruses such as dengue and influenza A from passing through the cellular lipid bilayer [13–16]. It has also been demonstrated to inhibit the infection of Ebola, HIV-1 (human immunodeficiency virus type I), and hepatitis C viruses [17]. Moreover, IFITM3 protein was found to block the S-protein dependent endocytosis of Middle East Respiratory Syndrome Coronavirus (MERS-CoV) [18], thus preventing the genetic material of the virus from entering the cell.

A previous study [19] has indicated that single nucleotide polymorphisms (SNPs) in the gene IFITM3 may reduce the antiviral activities of IFITM3, resulting in increased infection susceptibility and illness severity. The C-allele of the SNP rs12252 (c.-22T>C) was discovered to be strongly linked with the severity of H1N1 and H7N9 influenza A virus infections in Asians and Caucasians [20, 21].

## Subjects and methods

### Subjects

This is a case–control study in which 242 adult SARS-CoV-2 patients (group 1) were recruited from Main University Hospital, Alexandria University, Egypt, between 2/2022 and 8/2022, based on their presentation with SARS-CoV-2 typical symptoms such as fever, cough, and dyspnea, or who were admitted to the hospital with previously confirmed SARS-CoV-2 infection. All cases were confirmed in the laboratory with a positive result of SARS-CoV-2 infection from a real-time reverse transcription polymerase chain reaction analysis of pharyngeal and nasal swabs. Patients with a history of chronic viral infection (e.g., HCV, HBV, HIV) as well as malignant diseases were excluded from the study. The study comprised 187 healthy participants as a control (group 2) selected from cases and their accompanying relatives who were visiting the chemical pathology lab in another branch of Alexandria University Hospitals that is not receiving SARS-CoV-2 cases. Careful history was taken from these subjects for previous infection with SARS-cov-2, history of household or work contact with suspicious cases of SARS-CoV-2 in the previous 14 days, or possible symptoms of SARS-CoV-2 infection such as fever, sore throat, runny nose, dry or productive cough, shortness of breath or respiratory distress, fatigue, muscle or body aches, unexplained headache or altered mentality, new loss of taste or smell, nausea, vomiting, or diarrhea. These subjects were followed up in a second visit after 2 weeks to check for developing new symptoms. Subjects who did not apply for the second visit were considered non-compliant or had an infection and were not included in the study even though they had signed informed consent before.

Sample size was calculated using Power Analysis and Sample Size Software (PASS 2020) “NCSS, LLC. Kaysville, Utah, USA, ncss.com/software/pass”. The minimal total hypothesized sample size of 360 eligible patients (180 per group) is needed to investigate the Impact of the Single Nucleotide polymorphism of IL-27P28 rs153109 and IFITM3 rs12252 on the susceptibility and severity of COVID- 19 among Egyptians; taking into consideration 95% level of confidence, effect size of 0.7(hypothesized correlation coefficient) and standard deviation of 0.05, and power of 80% using Correlation analysis.

### Patients’ classification according to the severity of SARS-CoV-2 infection

According to the National Health Commission of China and Egyptian Ministry of Health (MOH) Guidelines [22, 23], we subdivided the patient groups into two subgroups: group A comprised mild/moderate cases (N = 42; 17.4%), and group B included severe/critical cases (N = 200; 82.6%).

The severity of SARS-CoV-2 was evaluated as follows:

1. Mild: clinical symptoms are minor, and a lung CT scan shows no pneumonia.
2. Moderate: fever, cough, and lung CT showing pneumonia.
3. Severe: respiratory distress (oxygen saturation (O2Sat) ≤ 93% at room air, respiratory rate > 30/min, and/or ratio of arterial oxygen partial pressure to fractional inspired oxygen ≤ 300 mmHg (PaO2/FIO2).
4. Critical: the aforementioned requirements plus respiratory failure requiring mechanical ventilation, shock, and/or organ failure other than lung and/or intensive care unit (ICU) hospitalization.

### Clinical and biochemical characteristics of the patients studied

The clinical characteristics of the patients were extracted from the medical records. These are:

1. Demographic variables and symptoms (age, sex, fever and diarrhea, degree of respiratory distress at presentation).
2. Routine biochemical investigations (complete blood count, serum urea, creatinine, sodium, potassium, alanine aminotransferase (ALT), aspartate aminotransferase (AST), international normalized ratio (INR) and C- reactive protein (CRP).
3. Comorbid conditions (hypertension, diabetes mellitus, cardiac disease, chronic bronchitis, chronic liver disease, chronic obstructive pulmonary disease, chronic renal disease, and cerebrovascular disease).

### Patient consent and ethical approval

Written informed consent was obtained from each participant incorporated in this study or first-degree relatives for patients who were admitted to the intensive care unit and/or on mechanical ventilators. In this study we followed the ethical guidelines of the Faculty of Pharmacy, Alexandria University, and obtained the approval of the Medical Ethics Committee, Faculty of Medicine, Alexandria University (serial number is 0107026; IRB number: 00012098).

### Genotyping

#### DNA extraction

Two milliliters of peripheral venous blood samples from all subjects were collected via venipuncture in ethylenediamine tetra acetic acid (EDTA) anticoagulated tubes for genotyping of IL27 rs153109 (-964A/G) and IFITM3 rs12252 (T>C).

Genomic DNA was extracted from blood samples using the QIAamp DNA Blood Mini kit, following the manufacturer’s instructions. The extracted DNA was analyzed on a 2% agarose gel to confirm the presence of genomic DNA. The DNA concentration in all samples was determined by using a Nanodrop 2000 (Thermo Scientific, USA). The DNA purity was evaluated using Kalckar’s formula (the OD260/OD280 ratio).

#### Genotyping of IL27P28 rs153109

**Fig. 1:**
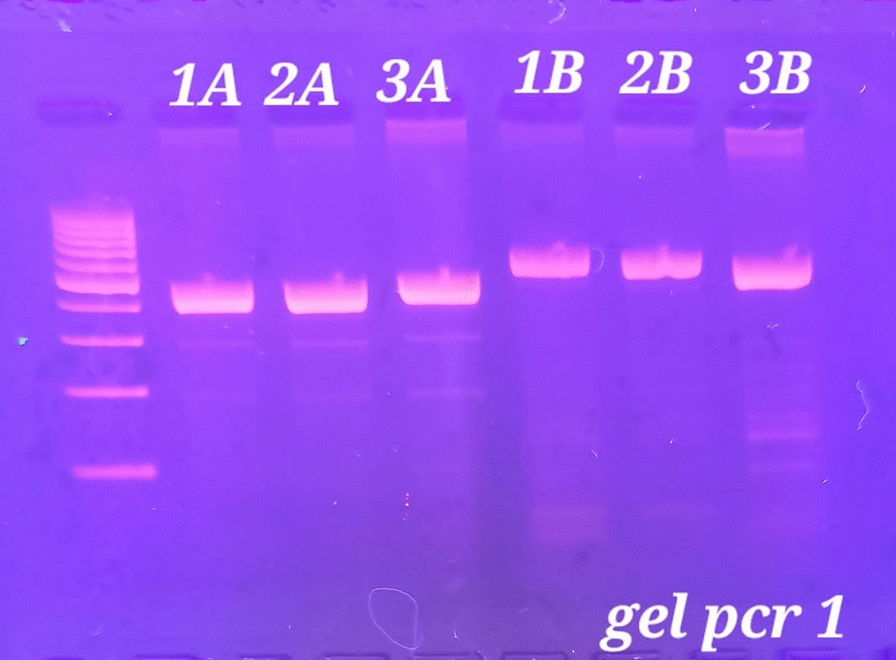
PCR products of IL27 rs153109 and IFITM3 rs12252 before addition of Xhol and MScI restriction enzymes, respectively. Lanes 1A,2A and 3A present the PCR product 468 bp of IL27p28 before adding Xhol restriction enzyme. Lanes 1B,2B and 3B present the PCR product 572 bp of IFITM3 before adding MScI restriction enzyme

**Fig. 2:**
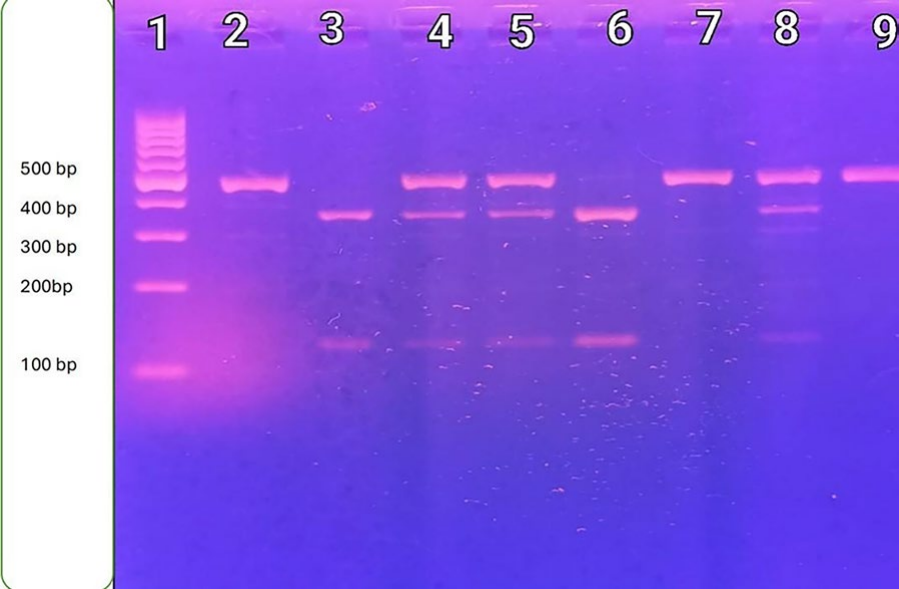
IL-27p28 rs153109 (964A/G) PCR product after digestion with Xho1 enzyme. Lane 1: shows Thermo Scientific Gene Ruler 100 bp DNA ladder. Lanes 2,7 and 9 show that A/A genotype is homozygous having A allele in the 2 copies of the chromosome, so the enzyme does not cut in both giving 2 pieces (each 2 have the same length so appear as 1 band 468 bp). Lanes 3 and 6 show G/G genotype is homozygous having G allele in the 2 copies of the chromosome so the enzyme cuts in both giving 4 pieces (each 2 have the same length so appear as 2 bands 347 bp, 121 bp). Lanes 4,5 and 8 show that A/G is heterozygous. Only one chromosome has the G allele, so the enzyme leaves one uncut 468 bp and cuts the other into 2 pieces (347,121) so A/G genotype appears as 3 bands (468 bp, 347 bp, 121 bp)

Single nucleotide polymorphism of IL27p28 rs153109 was detected by using a forward primer (5′-GGC TGT GCT GGA AGG GAG AC-3ʹ) and a reverse primer (5′-ATA TCT GGG ACC AGG GTT AGG-3ʹ) [24–27]. The polymerase chain reaction (PCR) settings were as follows: initial denaturation at 95 °C for 3 min followed by 40 cycles: denaturation at 95 °C for 30 s, annealing at 55 °C for 30 s, and extension at 72 °C for 1 min, culminating in a final extension at 72 °C for 10 min and cooling to 4 °C. The PCR product was cut by FastDigest XhoI restriction endonuclease at 37 °C for 5 min giving segments of 468 bp (A/A genotype), 347/121 bp (G/G genotype), and 468/347/121 bp (A/G genotype), which were visualised by UV light on agarose 2% gel electrophoresis, and ethidium bromide (1%) was stained [27] (Figs. 1, 2).

#### Genotyping of IFTIM3 rs12552

**Fig. 3:**
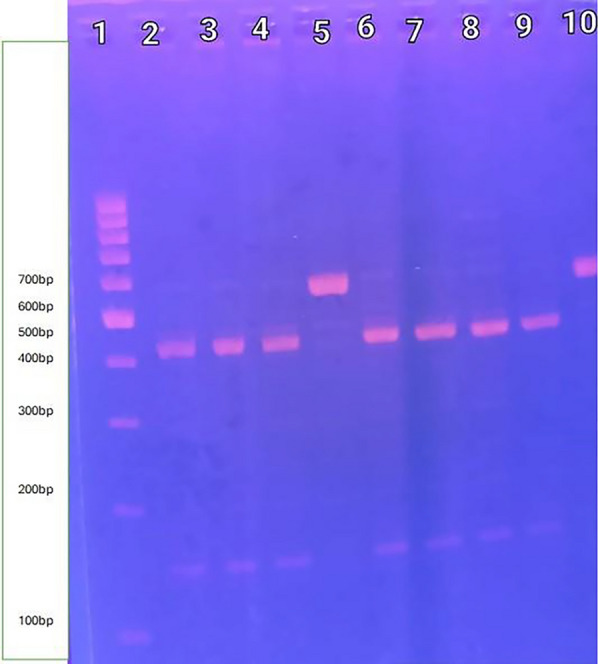
IFITM3 rs12252 PCR product after digestion with MScI enzyme (T/T genotype vs. C/C genotype). Lane 1: shows Thermo Scientific Gene Ruler 100 bp DNA ladder.Lanes 2,3,4,6, 7.8 and 9 show T/T genotype is homozygous having T allele in the 2 copies of the chromosome, so the enzyme cuts in both giving 4 pieces (each 2 have the same length so appear as 2 bands 426 bp, 146 bp). Lanes 5 and 10 show C/C genotype is homozygous having C allele in the 2 copies of the chromosome, so the enzyme does not cut in both giving 2 pieces (each 2 have the same length so appear as 1 band 572 bp)

**Fig. 4:**
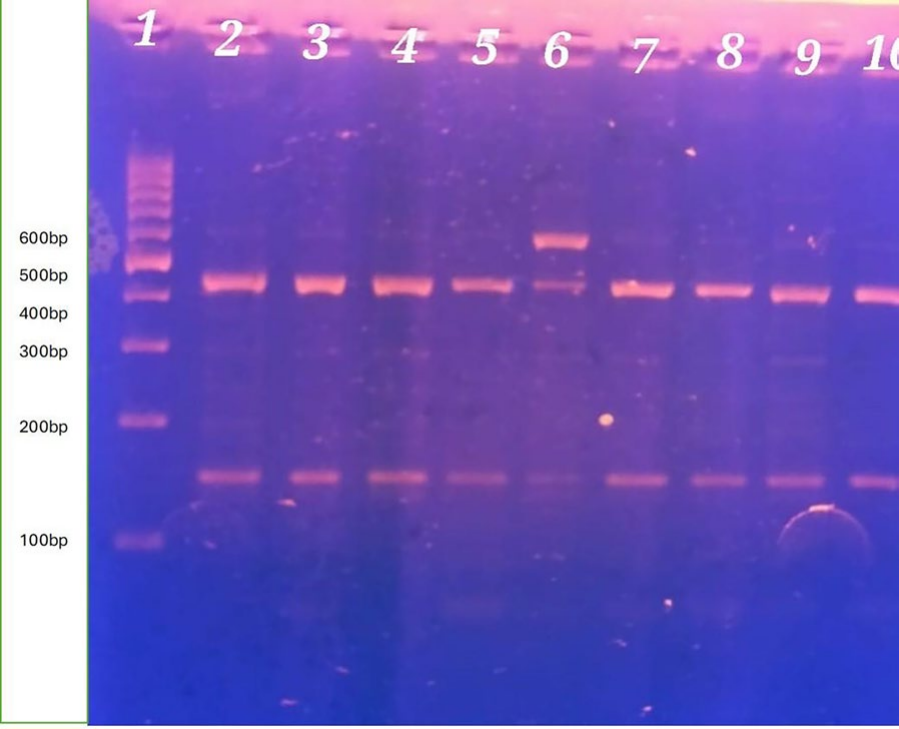
IFITM3 rs12252 PCR product after digestion with MScI enzyme (T/T genotype vs. C/T genotype). Lane 1: shows Thermo Scientific Gene Ruler 100 bp DNA ladder. Lanes 2,3,4,5,7.8,9 and 10 show T/T genotype is homozygous having T allele in the 2 copies of the chromosome, so the enzyme cuts in both giving 4 pieces (each 2 have the same length so appear as 2 bands 426 bp, 146 bp). Lane 6 shows C/T is heterozygous. Only one chromosome has the C allele so the enzyme leaves one uncut 572 bp and cuts the other into 2 pieces (426 bp, 146 bp) so C/T genotype appears as 3 bands

IFITM3 was genotyped using restriction fragment length polymorphism with the forward primer CAGGAAAAGGAAACTGTTGAGAACC(F) and the reverse primer CTCCTGGAGCCTCCTCCTCA(R) [28]. To ensure that IFITM3 is amplified rather than IFITM2, primers include 3′ penultimate base mismatches with IFITM3.The polymerase chain reaction (PCR) settings were as follows: initial denaturation at 95 °C for 3 min, followed by 40 cycles of denaturation at 95 °C for 30 s, annealing at 55 °C for 30 s, and extension at 72 °C for 1 min, culminating in a final extension at 72 °C for 10 min and cooling to 4 °C.MScI (New England Biolabs) was used to cut the PCR product in the presence of the T allele (wild type). Pieces having lengths of 572 bp (C/C genotype), 426/146 bp (T/T genotype), and 572/426/146 bp (C/T genotype) were visualised by UV light on agarose 2% gel electrophoresis, and ethidium bromide (1%) was stained [28] (Figs. 1, 3, 4).

### Statistical analysis

Sample size was calculated using Power Analysis and Sample Size Software (PASS 2020) “NCSS, LLC. Kaysville, Utah, USA, ncss.com/software/pass”. The minimal total hypothesized sample size of 360 eligible patients (180 per group) is needed to investigate the Impact of the Single Nucleotide polymorphism of IL-27P28 rs153109 and IFITM3 rs12252 on the susceptibility and severity of SARS-CoV-2 among Egyptians; taking into consideration 95% level of confidence, effect size of 0.7(hypothesized correlation coefficient) and standard deviation of 0.05, and power of 80% using Correlation analysis.

Data was entered into the computer and analyzed using IBM SPSS software version 20.0. (Armonk, NY: IBM Corp). Categorical data were presented as numbers and percentages. The Chi-square test was used to compare different groups. Alternatively, The Fisher Exact correction test was used when more than 20% of the cells have expected count less than 5. The Kolmogorov–Smirnov test was used to assess the normality of continuous data. Quantitative data were expressed as range (minimum and maximum), mean, standard deviation and median for normally distributed quantitative variables. A Student T-test was performed to compare two groups. In contrast, for quantitative variables that were not normally distributed, the Mann Whitney test was utilized to compare two groups. The population of the sample studied was investigated to determine its equilibrium with Hardy–Weinberg equation. A multivariate Logistic regression analysis was conducted using the Hosmer–Lemeshow test to identify the most independent factor influencing COVID-19 infection. The significance of the acquired results was determined at a 5% level.

## Results

### Demographic, clinical and biochemical characteristics of all studied patients

The study included 429 adult patients, 242 patients diagnosed with SARS-CoV-2 infection and 187 normal healthy participants as controls. In SARS-CoV-2 patients (group 1), 44.6% were males, while in healthy controls (group 2), 37.4% were males. There was no statistically significant difference between the two groups in mean sex (*P* = 0.134). SARS-CoV-2 patients were statistically significantly older than controls (*P* < 0.001). They had significantly higher platelets, total WBC, neutrophils, as well as significantly lower hemoglobin level, and lymphocytes than the control group (all *P* < 0.05) (Table 1).

**Table 1:** Comparison between SARS-CoV-2 cases and controls according to demographic and some biochemical characteristics

	Control (n = 187)	Cases (n = 242)	*p*
Sex			
Male	70 (37.4%)	108 (44.6%)	0.134
Female	117 (62.6%)	134 (55.4%)	
Age in years	35 (14–79)	56 (15–89)	< 0.001^*^
Hemoglobin (gm/dl)	13.85 ± 1.82	11.85 ± 2.19	< 0.001^*^
Platelets (× 10^9^/L)	200 (140–265)	211.5 (29–647)	0.006^*^
WBC (× 10^9^/L)	7.90 (5–10.3)	10.24 (1–45.8)	< 0.001^*^
Lymphocytes count	3 (1.5–4)	2 (0.16–39)	< 0.001^*^
Neutrophils count	5.80 (3.50–7)	6.95 (0.20–42.01)	< 0.001^*^

Qualitative data were described using number and percent and was compared using Chi square test, while normally quantitative data was expressed in Mean ± SD and was compared using student t-test, not normally distributed data was expressed in Median (Min.–Max.) and was compared using Mann Whitney test

SD, Standard deviation; *p*, *p* value for comparing the two studied groups; *, statistically significant at *p* ≤ 0.05; WBC, white blood cells

Concerning patients, the mean duration for hospital stay was 6.24 ± 3.96. While the mean days for ICU admission was 5.75 ± 4.28. Those who fully recovered were (88.0%) versus (12.0%) deaths. (18.6%) patients did not need oxygen supply while (6.6%) patients were on nasal canula, (2.1%) patients were on high flow nasal canula, (7.9%) patients were on oxygen masks, (43.4%) patients were on mask reservoir, and (21.5%) were on mechanical ventilation.

For all patients:(52.9%) patients did not have comorbidity, (20.2%) patients were diabetic, (31.8%) patients had hypertension, (9.9%) patients had chronic renal disease, (2.1%) patients had pulmonary disease (COPD, asthma, or lung fibrosis) and (11.2%) patients had cerebrovascular stroke, gallbladder disease, peptic ulcer, anemia, familial Mediterranean fever, ischemic heart disease, or obstructive sleep apnea (OSA) (Table 2).

**Table 2:** Distribution of clinical and biochemical characteristics among SARS-CoV-2-studied patients (n = 242)

	No. (%)
ICU admission	198 (81.8%)
Days stay in ICU (days)	
Mean ± SD	5.75 ± 4.28
Median (Min.–Max.)	5 (0–24)
Days stay in hospital	
Mean ± SD	6.24 ± 3.96
Median (Min.–Max.)	6 (0–24)
Full recovery	
Recovery	213 (88.0%)
Death	29 (12.0%)
Oxygen supply	
No	45 (18.6%)
Oxygen Mask	19 (7.9%)
Nasal Cannula	16 (6.6%)
High Flow Nasal Cannula	5 (2.1%)
Reservoir Mask	105 (43.4%)
Mechanical ventilation	52 (21.5%)
SaO2 (est) %	
Mean ± SD	83.11 ± 9.73
Median (Min.–Max.)	84.0 (50.0–99.0)
Comorbidity	
No comorbidity	128 (52.9%)
Diabetes mellitus	49 (20.2%)
Hypertension	77 (31.8%)
Chronic renal disease	24 (9.9)
Chronic Respiratory disease (COPD / asthma /lung fibrosis)	5 (2.1%)
Others#	27 (11.2%)
CRP	
Mean ± SD	52.17 ± 62.83
Median (Min.–Max.)	40.05 (0.30–618.0)
INR	
Mean ± SD	1.44 ± 0.32
Median (Min.–Max.)	1.40 (0.84–2.81)
ALT	
Mean ± SD	58.38 ± 56.31
Median (Min.–Max.)	51.0 (11.0–738.0)
AST	
Mean ± SD	49.82 ± 53.58
Median (Min.–Max.)	45.50 (10.0–795.0)
Blood urea	
Mean ± SD	63.94 ± 42.66
Median (Min.–Max.)	48.0 (15.0–274.0)
Serum Creatinine	
Mean ± SD	1.50 ± 1.04
Median (Min.–Max.)	1.30 (0.30–7.30)
Severity of COVID-19	
Mild/Moderate	42 (17.4%)
Severe/Critical	200 (82.6%)

SD, standard deviation; SaO2, oxygen saturation, CRP, C-reactive protein; INR, The international normalized ratio; ALT, serum alanine aminotransferase; AST, serum aspartate aminotransferase

Others#: cerebrovascular stroke or gallbladder disease or peptic ulcer or anemia or familial Mediterranean fever or ischemic heart disease or obstructive sleep apnea (OSA)

### Demographic, clinical and biochemical characteristics of mild/moderate versus severe critical patients

Patients with severe/critical SARS-CoV-2 infection (group B) had statistically significant older age, higher white blood cell (WBC) count, Neutrophils %, C-reactive protein (CRP), international normalized ratio (INR), serum alanine aminotransferase (ALT), serum aspartate aminotransferase (AST), blood urea, and serum creatinine, as well as significantly lower hemoglobin and lymphocyte % than mild/moderate cases (group A) (*P* < 0.05 for all). On the other hand, there is no significant difference between the two subgroups regarding sex (*P* = 0.073) and platelets (*P* = 0.782). While 51.5% of group B were associated with comorbidities, only 26.2% of the group A had comorbidities; this difference was statistically significant (*P* = 0.003). The presence of comorbidity (diabetes mellitus, hypertension, and kidney dysfunction) was significantly associated with group B (*P* = 0.009), (*P* = 0.001), and (*P* = 0.011) respectively (Table 3).

**Table 3:** Comparison between SARS-CoV-2 cases subgroup A (mild/moderate) (n = 42) and subgroup B (severe/critical) (n = 200) according to demographic, biochemical characteristics and comorbidities

	Severity		*p*
	Mild/Moderate (n = 42)	Severe/Critical (n = 200)	
Sex			
Male	24 (57.1%)	84 (42.0%)	0.073
Female	18 (42.9%)	116 (58.0%)	
Age in years	39.0 (20.0–80.0)	59.0 (15.0–89.0)	< 0.001^*^
Hemoglobin (gm/dl)	13.53 ± 1.54	11.50 ± 2.14	< 0.001^*^
Platelets (× 10^9^/L)	209.0 (120.0–567.0)	214.0 (29.0–647.0)	0.782
WBC (× 10^9^/L)	6.50 (1.0–16.0)	11.50 (2.60–45.80)	< 0.001^*^
Lymphocytes (%)	40.80 (7.30–80.0)	17.20 (2.0–85.20)	< 0.001^*^
Neutrophils (%)	46.80 (20.0–84.40)	75.20 (1.60–96.40)	< 0.001^*^
CRP	6.75 (0.50–255.0)	44.45 (0.30–618.0)	< 0.001^*^
INR	1.26 (0.84–2.0)	1.44 (0.93–2.81)	< 0.001^*^
ALT	43.0 (11.0–223.0)	54.0 (13.0–738.0)	< 0.001^*^
AST	34.50 (10.0–60.0)	46.50 (12.0–795.0)	< 0.001^*^
Blood urea	36.0 (15.0–150.0)	54.0 (19.0–274.0)	< 0.001^*^
Serum Creatinine	1.30 (0.30–1.80)	1.30 (0.30–7.30)	0.014^*^
Comorbidity			
Comorbidity	11 (26.2%)	103 (51.5%)	0.003^*^
Diabetes mellitus	2 (4.8%)	47 (23.5%)	0.009^*^
Hypertension	4 (9.5%)	73 (36.5%)	0.001^*^
Chronic renal disease	0 (0.0%)	24 (12.0%)	FE = 0.011^*^
Chronic respiratory disease (COPD/asthma/lung fibrosis)	1 (2.4%)	4 (2.0%)	FET = 1.000
Others#	7 (16.7%)	20 (10.0%)	FET = 0.277

Qualitative data were described using number and percent and was compared using Chi square test or Fisher Exact test, while normally quantitative data was expressed in Mean ± SD and was compared using student t-test, not normally distributed data was expressed in Median (Min.–Max.) and was compared using Mann Whitney test

SD, standard deviation; *p*, *p* value for comparing between Mild/Moderate and Severe/Critical;*, Statistically significant at *p* ≤ 0.05

CRP, C-reactive protein; INR, The international normalized ratio; ALT, serum alanine aminotransferase; AST, serum aspartate aminotransferase

Others#, cerebrovascular stroke or gallbladder disease or peptic ulcer or anemia or familial Mediterranean fever or ischemic heart disease or Obstructive sleep apnea (OSA)

### Comparison between control group (n = 187) and the patients’ group (n = 242) according to IL27 rs153109 (-964A>G) and IFITM3 rs12252 (c.-22T>C) SNP

The proportions of healthy participants and SARS-CoV-2 patients with different genotypes of IL27 rs153109 (-964A>G) were evaluated. GG genotype carriers in the SARS-CoV-2 patient group had a lesser proportion than in controls (6.2% vs. 12.8%), AG genotype carriers in the SARS-CoV-2 patients had a higher proportion than in controls (43.4% vs. 37.4%), AA genotype carriers in the SARS-CoV-2 patients had a higher proportion than in controls (50.4% vs. 49.7%) and all of these differences were statistically significant (*P* = 0.049), (AG+AA) genotypes carriers in the SARS-CoV-2 patients had a greater significant proportion than in controls (93.8% vs. 87.2%) (*p* = 0.018).G allele carriers in the SARS-CoV-2 patient group had a lesser proportion than in controls (27.9% vs. 31.6%), A allele Carriers in the SARS-CoV-2 cases had a greater proportion than in controls (72.1% vs.68.4%), although the difference was not statistically significant (*P* = 0.244) (Table 4).

**Table 4:** Comparison between the Control group and SARS-CoV-2 patients’ group according to IL27 rs153109 (-964A>G) and IFITM3 rs12252 (c.-22T>C) SNP

	Control (n = 187)	Cases (n = 242)	*p*
IL27 genotype			
G/G	24 (12.8%)	15 (6.2%)	0.049^*^
A/G	70 (37.4%)	105 (43.4%)	
A/A	93 (49.7%)	122 (50.4%)	
^**HW**^**χ**^**2**^** (p)**	3.325 (0.068)	1.496 (0.221)	
A/G+A/A	163 (87.2%)	227 (93.8%)	0.018^*^
Allele			
G	118 (31.6%)	135 (27.9%)	0.244
A	256 (68.4%)	349 (72.1%)	
IFITM3 genotype			
T/T	145 (77.5%)	165 (68.2%)	0.099
C/T	39 (20.9%)	71 (29.3%)	
C/C	3 (1.6%)	6 (2.5%)	
^**HW**^**χ**^**2**^** (p)**	0.041 (0.840)	0.255 (0.613)	
C/T+C/C	42 (22.5%)	77 (31.8%)	0.032^*^
Allele			
T	329 (88.0%)	401 (82.9%)	0.037^*^
C	45 (12.0%)	83 (17.1%)	

^HW^χ^2^, Chi square for goodness of fit for Hardy–Weinberg equilibrium (If *p* < 0.05-not consistent with HWE.); p, *p* value for comparing between the studied groups; *, statistically significant at *p* ≤ 0.05

The proportions of healthy participants and SARS-CoV-2 patients in different genotypes of IFITM3 rs12252 (c.-22T>C) were assessed. TT genotype carriers in the SARS-CoV-2 patient group had a lesser proportion than in controls (68.2% vs. 77.5%), CT genotype carriers in the SARS-CoV-2 cases had a greater proportion than in controls (29.3% vs. 20.9%), CC genotype carriers in the SARS-CoV-2 cases had a greater proportion than in controls (2.5% vs 1.6%) and all of these differences were not statistically significant (*P* = 0.099), (CT+CC) genotypes carriers in the SARS-CoV-2 patients had a greater significant proportion than in controls (31.8% vs. 22.5%) (*P* = 0.032). T allele carriers in the SARS-CoV-2 patient group had a lesser proportion than in controls (82.9% vs. 88%), C allele carriers in the SARS-CoV-2 cases had a significantly higher proportion than in controls (17.1% vs.12%) (*P* = 0.037). In both studied groups, the observed genotype frequencies of all gene variants studied followed the Hardy Weinberg equilibrium (*P* > 0.05 for all the SNP investigated) (Table 4).

### Logistic regression analysis for the association of IL27 rs153109 (-964A>G) and IFITM3 rs12252 (c.-22T>C) with the risk of SARS-CoV-2 infection

Showed statistical significance with an increased odds ratio. SARS-CoV-2 infection was positively associated with the AG genotype (OR = 2.099, 95% CI: 1.043–4.224, *P* = 0.038), the AA genotype (OR = 2.400, 95% CI: 1.177–4.894, *P* = 0.016) and the (AG+AA vs. GG) genotypes (OR = 2.228, 95% CI: 1.134–4.380, *P* = 0.020). The presence of the A-allele was not significantly associated with an increased SARS-CoV-2 infection risk (OR = 1.192, 95% CI: 0.887–1.600, *P* = 0.244) (Table 5).

**Table 5:** Association genotypes of IL27 rs153109 (-964A>G) and IFITM3 rs12252 (c.-22T>C) with SARS-CoV-2 susceptibility by logistic regression analysis

	Control® (n = 187)	Cases (n = 242)	Univariate		Adjusted Odd’s ratio	
			*p*	OR (LL–UL 95%C.I)	*p*	AOR (LL–UL 95%C.I)
IL27 genotype						
G/G®	24 (12.8%)	15 (6.2%)	1.000		1.000	
A/G	70 (37.4%)	105 (43.4%)	0.038^*^	2.099 (1.043–4.224)	0.013^*^	2.791 (1.237–6.295)
A/A	93 (49.7%)	122 (50.4%)	0.016^*^	2.400 (1.177–4.894)	0.033^*^	2.385 (1.075–5.291)
A/G+A/A	163 (87.2%)	227 (93.8%)	0.020^*^	2.228 (1.134–4.380)	0.017^*^	2.558 (1.186–5.517)
Allele						
G®	118 (31.6%)	135 (27.9%)	1.000		1.000	
A	256 (68.4%)	349 (72.1%)	0.244	1.192 (0.887–1.600)	0.220	1.240 (0.880–1.747)
IFITM3 genotype						
T/T®	145 (77.5%)	165 (68.2%)	1.000		1.000	
C/T	39 (20.9%)	71 (29.3%)	0.041^*^	1.600 (1.020–2.509)	0.188	1.419 (0.843–2.391)
C/C	3 (1.6%)	6 (2.5%)	0.431	1.758 (0.432–7.154)	0.350	2.132 (0.436–10.415)
C/T+C/C	42 (22.5%)	77 (31.8%)	0.033^*^	1.611 (1.041–2.495)	0.138	1.466 (0.884–2.432)
Allele						
T®	329 (88.0%)	401 (82.9%)	1.000		1.000	
C	45 (12.0%)	83 (17.1%)	0.038^*^	1.513 (1.024–2.237)	0.119	1.433 (0.912–2.252)

OR, odd’s ratio; ®, reference group; CI, confidence interval; LL, lower limit; UL, upper limit; p, *p* value for regression analysis for comparing with the reference genotype; *, statistically significant at *p* ≤ 0.05; #, adjusted with age

Univariate logistic regression analysis for the association of IFITM3 rs12252 (c.-22T>C) with the risk of SARS-CoV-2 infection showed statistical significance with an increased odds ratio. SARS-CoV-2 infection was positively associated with the CT genotype (OR = 1.600, 95% CI: 1.020–2.509, *P* = 0.041), the CC genotype (OR = 1.758, 95% CI: 0.432–7.154, *P* = 0.431), and the (CT + CC vs.TT) genotypes (OR = 1.611, 95% CI: 1.041–2.495, *P* = 0.033). The presence of the C-allele significantly associated with an increased the risk of SARS-CoV-2 infection (OR = 1.513, 95%CI: 1.024–2.237, *P* = 0.038) (Table 5).

After adjusting for age, SARS-CoV-2 infection was positively associated only with the IL27 rs153109 AG genotype (OR = 2.791, 95% CI: 1.237–6.295, *P* = 0.013), the AA genotype (OR = 2.385, 95%CI: 1.075–5.291, *P* = 0.033), and the (AG+AA vs. GG) genotypes (OR = 2.558, 95% CI: 1.186–5.517, *P* = 0.017).On the other hand analysis after adjusting for age for IFITM3 rs12252 (c.-22T>C) CT genotype (OR = 1.419, 95%CI: 0.843–2.391, *P* = 0.188), the CC genotype (OR = 2.132, 95%CI: 0.436–10.415, *P* = 0.350) and the (C/T+C/C vs. TT) genotypes (OR = 1.466, 95% CI: 0.884–2.432, *P* = 0.138) did not show statistical significance (Table 5).

Multivariate analysis showed that the advanced age (OR:1.081, 95% CI: 1.063–1.099, *P* < 0.001) and IL27 rs153109 (-964A>G) [A/G+A/A] genotype (OR: 2.615, 95% CI: 1.203–5.687, *P* = 0.015) were statistically significant independent predictors for SARS-CoV-2 susceptibility, while IFITM3 had no statistically significant difference (OR:1.488, 95% CI: 0.895–2.473, *P* = 0.125) (Table 6).

**Table 6:** Univariate and Multivariate logistic regression analysis for the different parameters affecting the susceptibility of SARS-CoV-2 infection

	Univariate		^#^Multivariate	
	*P*	OR (LL–UL 95%C.I)	*p*	OR (LL–UL 95%C.I)
Gender				
Female®	1.000			
Male	0.134	1.347 (0.912–1.989)		
Age in years	< 0.001^*^	1.080 (1.063–1.097)	< 0.001^*^	1.081 (1.063–1.099)
IL27 genotype				
G/G®	1.000		1.000	
A/G+A/A	0.020^*^	2.228 (1.134–4.380)	0.015^*^	2.615 (1.203–5.687)
IFITM3 genotype				
T/T®	1.000		1.000	
C/T+C/C	0.033^*^	1.611 (1.041–2.495)	0.125	1.488 (0.895–2.473)

(n = 242 vs. 187) Hosmer and Lemeshow Test (χ^2^ = 2.689; *p* = 0.952) OR, odd’s ratio; C.I, confidence interval; LL, lower limit; UL, upper limit ®, Reference group; #, All variables with *p* < 0.05 was included in the multivariate; *, Statistically significant at *p* ≤ 0.05

### Allele combination analysis of IL27 rs153109 (-964A>G) and IFITM3 rs12252 (c.-22T>C)

Allele combination analysis of IL27 rs153109 (-964A>G) and IFITM3 rs12252 (c.-22T>C) variants has been performed to detect the combined allele that could be risk for SARS-CoV-2. When the allele combination G-T, consisting of the 2 alleles: minor allele G from IL27 rs153109 and major allele T from IFITM3 rs12252, was considered as reference, the allele combination A from IL27 rs153109 and minor allele C from IFITM3 rs12252 were associated with an increased risk of SARS-CoV-2 (OR = 1.667, 95% CI: 1.046–2.657, *P* = 0.031). After adjusting for age, AC haplotype did not show a significant association with risk for SARS-CoV-2 (Table 7).

**Table 7:** Allele combination analysis of IL27 rs153109 (-964A>G) and IFITM3 rs12252 (c.-22T>C) variants among the Control group and SARS-CoV-2 patients’ group (Haplotype)

	Control® (n = 374)	Cases (n = 484)	*p*	OR (LL–UL 95%C.I)	*p*	AOR^#^ (LL–UL 95%C.I)
Haplotype						
GT®	20 (23.8%)	104 (26.0%)		1.000		1.000
GC	1 (1.2%)	10 (2.5%)	0.336	1.656 (0.593–4.624)	0.747	1.206 (0.387–3.762)
AT	51 (60.7%)	226 (56.5%)	0.370	1.153 (0.845–1.574)	0.371	1.180 (0.821–1.695)
AC	12 (14.3%)	60 (15.0%)	0.031^*^	1.667 (1.046–2.657)	0.061	1.678 (0.976–2.883)

OR, odd’s ratio; AOR, adjusted odd’s ratio; CI, confidence interval; LL, lower limit; UL, upper limit; *p*: *p* value for Univariate regression analysis for comparing with the reference genotype; ®Reference group; *, statistically significant at *p* ≤ 0.05; #, adjusted with age

### Association genotypes of IL27 rs153109 (-964A>G) and IFITM3 rs12252 (c.-22T>C) with severity of SARS-CoV-2 disease in all patients group

Single nucleotide polymorphism of IL27 rs153109 (-964A>G) and IFITM3 rs12252 (c.-22T>C) did not show statistically significant linkage with the severity of SARS-CoV-2 disease (*p* = 0.16) and (*p* = 0.237) respectively (Table 8).

**Table 8:** Association genotypes of IL27 rs153109 (-964A>G) and IFITM3 rs12252 (c.- 22T>C) with severity of SARS-CoV-2 disease

	Mild/Moderate (n = 42)	Severe/Critical (n = 200)	*p*
IL27 genotype			
G/G	0 (0.0%)	15 (7.5%)	0.160
A/G	21 (50.0%)	84 (42.0%)	
A/A	21 (50.0%)	101 (50.5%)	
A/G+A/A	42 (100.0%)	185 (92.5%)	^FE^*p* = 0.080
Allele			
G	21 (25.0%)	114 (28.5%)	0.516
A	63 (75.0%)	286 (71.5%)	
IFITM3 genotype			
T/T	31 (73.8%)	134 (67.0%)	0.237
C/T	9 (21.4%)	62 (31.0%)	
C/C	2 (4.8%)	4 (2.0%)	
C/T+C/C	11 (26.2%)	66 (33.0%)	0.389
Allele			
T	71 (84.5%)	330 (82.5%)	0.665
C	13 (15.5%)	70 (17.5%)	

FET, fisher exact test; *p*, *p* value for comparing between Mild/Moderate and Severe/Critical

### Allele combinations of IL27 rs153109 (-964A>G) and IFITM3 rs12252 (c.-22T>C) in mild/moderate group and severe/critical group

There was no significant difference detected between all the combined alleles GC, AT and AC of IL27 rs153109 (-964A>G) and IFITM3 rs12252 (c.-22T>C) and the severity of SARS-CoV-2 disease (Table 9).

**Table 9:** Comparison between the Mild/Moderate group and Severe/Critical group according to combinations of IL27 rs153109 (-964A>G) and IFITM3 rs12252 (c.-22T>C) (Haplotype)

	Mild/Moderate (n = 84)	Severe/Critical (n = 400)	*p*	OR (LL–UL 95%C.I)
Haplotype				
GT®	20 (23.8%)	104 (26.0%)		1.000
GC	1 (1.2%)	10 (2.5%)	0.544	1.923 (0.233–15.871)
AT	51 (60.7%)	226 (56.5%)	0.580	0.852 (0.483–1.502)
AC	12 (14.3%)	60 (15.0%)	0.922	0.962 (0.439–2.104)

OR, odd’s ratio; ®, reference group; CI, confidence interval; LL, Lower limit; UL, upper limit; *p*, *p* value for Univariate regression analysis for comparing with the reference genotype

## Discussion

For the first time, we found a novel association between IL27 rs153109 (A>G) and the susceptibility to SARS-CoV-2 infection among the Egyptian population, with AA and AG being at high risk. However, it was not significantly associated with the severity of the disease. With respect to IFITM3 rs12252 (T>C) CT, (CT+CC vs. TT) and C allele had a significant association with susceptibility to SARS-CoV-2 infection, but after adjusting for the risk factor (advanced age), it did not show a significant association with susceptibility to SARS-CoV-2 infection among the Egyptian population. Furthermore, IFITM3 rs12252 (T>C) did not show a significant association with the severity of the disease.

SARS-CoV-2 has emerged as a global danger to public health. Infected people can be asymptomatic or experience mild symptoms such as dry cough and diarrhea to severe symptoms such as pneumonia, respiratory distress, and even death. In Egypt, 516,023 confirmed cases and 24,830 deaths were reported to WHO until 16 January 2025 [3]. Yet, no studies have examined the association between IL27 rs153109 (-964A/G) and both the risk and the severity of SARS-CoV-2 as well as, the association between the IFITM3 rs12252 SNP and both the susceptibility and the severity of SARS-CoV-2 has not been done on Egyptians but other population. So, our aim in this study was to investigate the impact of the single nucleotide polymorphisms of IL-27P28 rs153109 and IFITM3 rs12252 on the susceptibility and the severity of SARS-CoV-2 among Egyptians. Our study included 242 Egyptian patients, recruited from Main University Hospital, Alexandria University, Alexandria, Egypt, and 187 Egyptian healthy controls. We found that IL27P28 rs153109 AG and AA genotypes of IL27 may be associated with the susceptibility of SARS-CoV-2 infection (*p* = 0.017) but not the severity (*p* = 0.08). Concerning the IFITM3 rs12252 SNP, we could not confirm its influence on either susceptibility or the severity of SARS-CoV-2 (*p* = 0.099) and (*p* = 0.237) respectively, in the Egyptian population.

In our study, the severe/critical patients were older and had a higher prevalence of comorbidities than mild/moderate patients, and the presence of these comorbidities was significantly associated with a more progressive course of disease, which is defined in other studies, such as Zayed NE et al. [23] who investigated determinants of severity in 202 SARS-CoV-2 Egyptian patients and discovered that older people with diabetes mellitus, hypertension (HTN), chronic respiratory illness, and ischemic heart disease (IHD) were considerably more likely to develop severe SARS-CoV-2 disease. He et al. [29] discovered that patients with chronic kidney illness developed severe SARS-CoV-2 disease. Furthermore, Du RH et al. [30] discovered that patients over 65 years old had cardiovascular and cerebrovascular comorbidities associated with severe SARS-CoV-2 illnesses. A recent meta-analysis summarised the linked host-related risk variables and indicated that severe SARS-CoV-2 patients were more likely to be older with associated various cardiovascular and respiratory comorbidities, which was explained by poor immune function [31].

Our study also showed that severe/critical patients had a higher significant level of WBC, Neutrophils %, CRP, ALT, AST, blood urea, serum creatinine, and INR than mild/moderate patients but lower levels of hemoglobin and lymphocyte % compared to mild/moderate patients, which agrees with previous studies [32–35] revealing that these severe/critical patients had more severe inflammatory responses besides having renal and hepatic affections.

IL27 has attracted substantial attention due to research suggesting that IL27 inhibits virus replication, including hepatitis B virus (HBV) [36] and HIV-1 [37]. Hepatitis C virus (HCV) [38], influenza A virus (IAV) [39] and cytomegalovirus (CMV) [40]. Furthermore, IL27 rs153109** (**-964A/G) has been related to many diseases such as inflammatory bowel disease and asthma [10, 41].

Zamani et al. [42] stated that severe SARS-CoV-2 patients had significantly greater levels of IL-27 compared to non-severe SARS-CoV-2 patients and healthy subjects among Iranians. They suspected that elevated levels of IL-27 in severe SARS-CoV-2 are linked to illness recovery, possibly due to its effect on generating antiviral proteins and stimulating some immune cells that play critical roles in viral infections. Besides, severe cases requiring ICU therapy showed significant reduction in IL-27 levels while SARS-CoV-2 survivors had significantly higher levels of IL-27 compared to those who died from the virus*.* This is in line with our study investigating IL27 rs153109 (-964A/G) among all the studied groups. We found that IL27 rs153109 (-964A/G) AG and AA genotypes carriers in SARS-CoV-2 patients had greater proportions than in controls (both *p* < 0.05). Logistic regression analysis for the association of IL27 rs153109 (-964A/G) with SARS-CoV-2 infection susceptibility showed statistical significance with a higher odds ratio. After adjusting for the risk factor (advanced age), the association of IL27 rs153109 (-964A/G) with SARS-CoV-2 infection susceptibility remained statistically significant with a higher odds ratio, which means that AG and AA can cause high susceptibility to SARS-CoV-2 while GG made low susceptibility to the disease. On the other hand, IL27 rs153109 (-964A/G) AG and AA genotypes did not show statistical significance associated with the severity of SARS-CoV-2 disease. To our knowledge, no available studies until now have been conducted on IL27 rs153109 (-964A/G) regarding susceptibility or severity of SARS-CoV-2 infection.

With respect to IFITM3, it has been documented to inhibit the infection of several viruses, including influenza A virus and SARS-CoV [43].This protein’s antiviral effect is achieved by its dimerization on endo-lysosomal membranes, which makes fusion pore formation energetically unfavorable, inhibiting hemifusion and viral escape into the cytoplasm [44]. The association between the IFITM3 rs12252 (T>C) SNP and either the susceptibility or severity of SARS-CoV-2 disease has been a debate. A systematic review and meta-analysis of five studies on Germany, Spain, China, and Saudi Arabian populations unveiled a statistically significant association between the polymorphism IFITM3 rs12252 (T>C) and susceptibility to SARS-CoV-2 infection. However, there was no significant association between IFITM3 rs12252 (T>C) and SARS-CoV-2 severity [45]. Another meta-analysis included five studies suggested that the IFTM3 rs12252 CC genotype was significantly linked to a higher risk of severe SARS-CoV-2 and mortality in the Chinese population, while the IFTM3 rs12252 C allele may be associated with increased risk of SARS-CoV-2 mortality in the Caucasian population [46]. Zhang et al. [47] also found a significantly greater prevalence of rs12252 C-allele carriers in patients with severe SARS-CoV-2 compared to patients with mild SARS-CoV-2. Recently, it was revealed in a Spanish cohort [13] that C-allele carriers of the SNP rs12252 have a twofold greater risk of SARS-CoV-2 infection compared to a control group obtained before the pandemic. On the contrary, a study conducted in a German cohort did not detect an association between IFITM3 rs12252 (T>C) and both SARS-CoV-2 infection susceptibility and severity [48].

Our results showed that IFITM3 rs12252 (T>C) CT and CC genotype carriers in SARS-CoV-2 patients had significantly greater proportions than in controls (*p* = 0.032) with the C-allele in SARS-CoV-2 patients being in greater proportions than in controls (*p* = 0.037). Logistic regression analysis for the association of IFITM3 rs12252 (T>C) with SARS-CoV-2 infection susceptibility showed statistical significance with a higher odds ratio, but after adjusting for the risk factor (advanced age), the association of IFITM3 rs12252 (T>C) with SARS-CoV-2 infection susceptibility showed not statistical significance association between IFITM3 rs12252 (T>C) CT and CC genotypes, and C-allele and risk of SARS-CoV-2 susceptibility. Concerning the severity of SARS-CoV-2 disease, IFITM3 rs12252 (T>C) CT and CC genotypes, and C-allele did not show statistical significance associated with the severity of SARS-CoV-2 disease, which agrees with Schönfelder et al. [48], who did not find an association for IFITM3 rs12252 (T>C) with SARS-CoV-2 infection susceptibility or severity in a German cohort and also agrees with the systematic review and meta-analysis [45], which unveiled that there was no significant association between IFITM3 rs12252 (T>C) and SARS-CoV-2 severity.

Finally, our study did not find a significant association between the allele combination A-allele from IL27 rs153109 **(**-964A>G) and C-allele from IFITM3 rs12252 (T>C) and either the susceptibility or severity of SARS-CoV-2 disease among the Egyptian patients.

## Conclusion

IL27P28 rs153109 AG and AA genotypes of IL27 may be associated with the susceptibility of SARS-CoV-2 infection but not the severity. With respect to IFITM3 rs12252 (T>C) CT, (CT+CC vs. TT) and C allele had a significant association with susceptibility to SARS-CoV-2 infection, but after adjusting for the risk factor (advanced age), it did not show a significant association with susceptibility to SARS-CoV-2 infection among the Egyptian population. Furthermore, IFITM3 rs12252 (T>C) did not show a significant association with the severity of the disease.

Since many of our participants were not vaccinated, we were unable to assess the role of vaccination on patient outcomes or susceptibility to infection, so we recommend that future studies include vaccination criteria.

## Data Availability

The data presented in this study are available upon request from the corresponding author. The data are not publicly available due to privacy and ethical reasons.

## Abbreviations

ALT: Serum alanine aminotransferase
AST: Serum aspartate aminotransferase
COVID-19: Coronavirus disease 2019
CRP: C-reactive protein
HBV: Hepatitis B virus
HCV: Hepatitis C virus
HIV: Human immunodeficiency virus
IFITM3: Interferon-induced transmembrane protein 3
IL-27: Interleukin27
INR: The international normalized ratio
MERS-CoV: Middle East respiratory syndrome coronavirus
PCR: Polymerase chain reaction
SARS-CoV: Severe acute respiratory syndrome coronavirus
SNP: Single Nucleotide polymorphism
WBC: Weight blood cell
